# Efficacy of adding dexmedetomidine as adjuvant with bupivacaine in ultrasound-guided intermediate cervical plexus block for thyroidectomy surgery: randomized controlled study

**DOI:** 10.1186/s12871-025-02990-7

**Published:** 2025-03-25

**Authors:** Mamdouh Mahmoud Mostafa, Reham M. Gamal, Aya M. Ahmed Baiomy, Mohamed Elsayed Hassan, Jehan Mohamed Kamal, Thabet TS, Tamer A. Kotb, Mai M. Elrawas

**Affiliations:** Department of Anesthesia, SICU and pain Management, National Cancer Institute, Cairo University, Cairo, Egypt

**Keywords:** Bupivacaine, Intermediate cervical plexus block, Dexmedetomidine, Efficacy

## Abstract

**Background:**

One important aspect of a successful thyroidectomy recovery is the level of pain postoperatively. This research aimed to determine the effectiveness of an ultrasound-guided intermediate cervical plexus block (CPB) for thyroidectomy with dexmedetomidine added as an adjuvant to bupivacaine. The primary outcome was the duration of analgesia defined as the time till the first request for rescue analgesia. The secondary outcomes were the total amount of fentanyl consumed intraoperatively, total patient’s opioids requirements within 24 h postoperative, VAS, and complications.

**Methods:**

This randomized controlled double-blinded study included 60 patients aged 18 to 60 years, all of whom underwent thyroidectomy for thyroid cancer. Patients were randomly allocated into two equal groups, the B Group (*n* = 30) received bilateral intermediate CPB, with 20 ml bupivacaine 0.25%, and the DB Group (*n* = 30) received bilateral intermediate CPB with 20 ml of bupivacaine 0.25% plus 1 µg/kg dexmedetomidine.

**Results:**

The DB Group showed a significantly longer duration of analgesia (*p* < 0.001), significantly less total intraoperative fentanyl consumption (*p* = 0.005), and significantly less total postoperative morphine consumption (*p* < 0.001). Also, postoperative pain scores, heart rate, and mean arterial pressure were significantly lower in the DB group than in the B Group and sometimes points.

**Conclusions:**

The addition of dexmedetomidine to bupivacaine in ultrasound-guided intermediate CPB for thyroidectomy significantly prolonged analgesia and reduced postoperative opioid consumption.

**Supplementary Information:**

The online version contains supplementary material available at 10.1186/s12871-025-02990-7.

## Introduction

Most thyroid surgeries require a brief hospitalization but are accompanied by mild to moderate postoperative pain. Nevertheless, certain individuals may need opioid analgesia throughout the postoperative period. While opioids remain the benchmark treatment for pain relief after surgery, the administration of high doses is associated with a range of side effects that differ in severity, including nausea, vomiting, dizziness, constipation, respiratory depression, hypoventilation, and disturbances in sleep-related breathing [1]. Consequently, alternative strategies are advisable to ensure that analgesia is both effective and appropriate. Suppressing central sensitization can reduce postoperative pain and opioid usage through peripheral nerve block [2].

The cervical plexus block (CPB) is an efficient method for relieving pain during surgical procedures involving the head and neck [3]. It delivers strong anesthesia and pain relief locally in the area around the C2 to C4 nerve roots [4]. Innovative methods of ultrasound-guided superficial, intermediate, and deep CPB have been developed, showing enhanced effectiveness and safety in several fields including thyroid surgery [5]. Intermediate CPB is preferred over both superficial and deep techniques as it demonstrated highly effective analgesia in neck procedures, including carotid endarterectomy and thyroid interventions [6, 7].

It is appropriate to use adjuvants such as alpha-2 agonists, neostigmine, or magnesium since local anesthetic drugs have a relatively short duration of action. Dexmedetomidine is an agonist that targets alpha-2 adrenoceptors (α2-AR) with high selectivity, exhibiting tenfold greater specificity than clonidine. It induces analgesia, anxiolysis, and sedation in a dose-dependent manner, without causing respiratory depression, at both the spinal and supraspinal sites [8]. Dexmedetomidine elevates the effects of anesthesia produced by other anesthetic medications, promotes relaxation of the nervous system around the time of surgery, and decreases blood pressure [9].

The renewed interest in using α2-agonists alongside local anesthetics is intended to enhance the duration of the effects of these blocks. Consequently, this research was done to investigate the effectiveness of adding dexmedetomidine to bupivacaine in ultrasound-guided intermediate CPB for thyroidectomy.

## Methods

This randomized double-blind controlled trial involved 60 patients aged 18 to 60, classified as American Society of Anesthesiologists (ASA) physical status II and III and scheduled for thyroidectomy for thyroid cancer under general anesthesia (GA). The investigation was conducted from September 2022 to March 2024, following authorization from the Ethical Committee of Cairo University Hospitals (Approval code: AP2211-301-045) and registration at clinical trial gov. (ID: NCT05814744, and the date is 2024-04-05). Consent was obtained from patients via written reports. Exclusion criteria included serious adverse reactions to dexmedetomidine or local anesthetics, a diagnosed mental illness, a retro-sternal goiter, changed anatomical landmarks, a localized infection at the block site, or coagulopathy with an international normalized ratio (INR) ≥ 1.6.

### Randomization and blinding

The allocation of patients was done randomly using computer-generated random numbers, and the codes for each patient were stored in a sealed, opaque envelope. Two equal groups of patients were randomly assigned at a 1:1 ratio. Group A (*n* = 30) received bilateral intermediate CPB, with 20 ml bupivacaine 0.25% after general anesthesia (GA) induction. Group B received bilateral intermediate CPB, 20 ml of bupivacaine 0.25% with 1 µg/kg dexmedetomidine after GA induction. Patients and investigators were blinded to group allocation. An additional anesthesiologist who did not take part in any of the other aspects of the research conducted the preparation of the research solutions.

All patients underwent a clinical examination, routine laboratory investigations, and a medical and surgical history. These investigations encompassed a complete blood count, coagulation profile, liver and kidney functions, an electrocardiogram for patients over the age of 40, and any additional necessary investigations for high-risk patients. Postoperative pain assessment was considered with each patient using the Visual Analogue Scale (VAS). VAS (0 denotes “no pain,” while 10 denotes “the most horrible pain conceivable”).

### Intraoperative care

An Intravenous (IV) cannula was fixed for each patient and 0.02 mg/kg midazolam was administered. The ECG, NIBP, pulse oximeter, temperature probe, and capnogram were utilized to continuously monitor all patients during the surgery. An IV dose of 2 mg/kg propofol and 1 µg/kg fentanyl were delivered to induce GA, and 0.5 mg/kg rocuronium was administered to facilitate tracheal intubation. The B Group received bilateral intermediate CPB after induction of GA, with 20 ml bupivacaine 0.25% after GA induction. The DB Group received bilateral intermediate CPB, 20 ml of bupivacaine 0.25% with 1 µg/kg dexmedetomidine (precedex^®^). Anesthesia was maintained by inhaling sevoflurane in oxygen-enriched air (FiO_2_ = 50%) with a MAC of 2-2.5, and 0.1 mg/kg of rocuronium was administered IV as needed. One gram of IV paracetamol was given to every patient.

If the mean arterial blood pressure or heart rate exhibited a rise of more than 20% from their initial readings, further 0.5 µg/kg bolus doses of fentanyl were administered.

### Ultrasound-guided intermediate CPB

A complete set of aseptic precautions was implemented during the block. The head swiveled to one side throughout the block, which was administered in the supine position. The relative length and position of the sternocleidomastoid (SCM) were evaluated by exposing the neck and upper torso. A Sonosite M-Turbo C 04TRGD ultrasound apparatus was used with a 10–12 MHz linear array probe (HFL38). The transducer was put on the side of the neck aligned with the cricoid cartilage or midway above the SCM. After the SCM was located, the transducer was repositioned until its tapering back edge was centered on the screen. At this point, the brachial plexus groove was located, which is formed by the front and middle scalene muscles. The prevertebral fascia was identified as a cluster of tiny hypoechoic nodules above the interscalene groove. These nodules represent the cervical plexus. To block the intermediate cervical plexus nerve, a punch was used to perforate the investing layer and prevertebral fascia of the deep cervical fascia. To verify that the injection site was correct, 1–2 mL of LA was administered after negative aspiration. The remainder of LA was administered to envelop the plexus.

### Postoperative care

Neuromuscular paralysis was reversed by a combination of 0.05 mg/kg neostigmine and 0.02 mg/kg atropine. Extubation was done once the airway reflexes were recovered. The patients were transferred to the post-anesthesia care unit (PACU) to monitor pain, mean arterial blood pressure (MAP), and HR at the time of admission and 2, 4, 6, 12, and 24 h after the procedure. Ramsay sedation score was used to evaluate the depth of sedation immediately after extubation and after 30 min. The total morphine dosage in 24 h was documented. Acetaminophen 1 gm IV was administered to each patient every eight hours. If the pain score exceeded 3, an IV bolus of 3 mg of morphine was used as a rescue analgesic. Hypotension (MAP reduction by 20% from basal) was treated with IV fluids, and bradycardia (Heart rate (HR) reduction by 20% from basal) was treated with IV atropine 0.02 mg/kg, respiratory depression (the SpO2 < 95% and need O2 supplementation), and postoperative nausea and vomiting (PONV).

The primary outcome was the duration of analgesia defined as the time till the first request for rescue analgesia. The secondary outcomes were the total amount of fentanyl consumed intraoperatively, total patient’s opioids requirements within 24 h postoperative, VAS, and complications.

### Sample size calculation

According to multiple previous clinical investigations, we expected a large effect size of 0.86 for the impact of adding dexmedetomidine as an adjuvant to ropivacaine on the duration of analgesia. To achieve a power of 90% and a level of significance of 5%, for detecting a true difference in means between the two groups, we need a sample of 30 patients in each group. G*Power 3.1.9.2 (Universitat Kiel, Germany) was employed to calculate the sample size.

### Statistical analysis

We used SPSS v26 (IBM Inc., Chicago, IL, USA) for our statistical analysis. Through the use of histograms and the Shapiro-Wilks test, the normality of the data distribution was calculated. The two groups were examined employing an unpaired Student’s t-test for quantitative parametric variables, which were presented in the form of the mean and standard deviation (SD). As a representation of quantitative non-parametric data, the Mann-Whitney test was used on the median and interquartile range (IQR). The frequencies and percentages of qualitative categories were examined using either the Chi-square test or Fisher’s exact test, depending on the situation. Statistical significance was established by a two-tailed P value of less than 0.05.

## Results

**Fig. 1:**
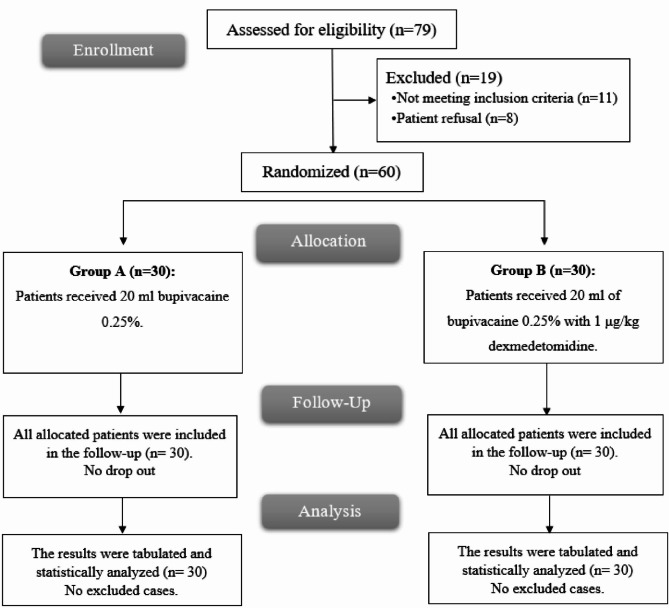
CONSORT flowchart of the enrolled patients

Seventy-nine patients were considered for inclusion, 8 declined to participate and 11 did not fulfill the inclusion criteria. The remaining 60 patients were finally analyzed (Fig. 1). There were no statistically significant differences between the two groups in age, sex, height, weight, BMI, ASA physical status, and surgery duration (Table 1).

**Table 1:** Demographic data and duration of surgery of the two groups

		B Group (*n* = 30)	DB Group (*n* = 30)	*p*-value
**Age (years)**		41.9 ± 9.2	44.6 ± 7.8	0.230
**Sex**	**Male**	11 (36.67%)	9 (30%)	0.584
	**Female**	19 (63.33%)	21 (70%)	
**Weight (kg)**		75.6 ± 12.3	79.2 ± 11.0	0.230
**Height (cm)**		167 ± 7	166 ± 7	0.487
**Body mass index (kg/m** ^**2**^ **)**		26.9 ± 3.72	28.76 ± 4.29	0.078
**ASA physical status**	**II**	21 (70.0%)	19 (63.3%)	0.584
	**III**	9 (30.0%)	11 (36.7%)	
**Duration of surgery (min)**		91 ± 18	93 ± 17	0.661

Data is presented as mean ± SD or frequency (%). BMI: Body mass index. ASA: American society of anesthesiologists

The duration of analgesia was significantly longer in the DB Group than in the B Group (*p* < 0.001). The total intraoperative fentanyl and postoperative morphine used, as well as the VAS scores at 6 and 12 h, were significantly lower in the DB Group than in the B Group. VAS scores were comparable in the two groups on arrival to the PACU and after 2, 4, and 24 h (Table 2).

**Table 2:** Analgesic efficacy of the two groups

		B Group (*n* = 30)	DB Group (*n* = 30)	95% CI	*p*-value
**Duration of analgesia (h)**		7.2 ± 0.9	10.2 ± 1.6	-3.62: -2.25	< 0.001*
**Total intraoperative fentanyl consumption (µg)**		102.2 ± 15.2	86.9 ± 23.7	---	0.005*
**Total postoperative morphine consumed (mg)**		7.0 ± 1.6	5.4 ± 1.5	0.8: 2.4	< 0.001*
**Visual analog scale score**	At arrival	1 (0–1)	0 (0–1)	---	0.609
	2 h	1 (1–2)	1 (1–1)		0.054
	4 h	2 (2–3)	2 (2–3)		0.225
	6 h	3 (2–4)	2 (1–2)		0.007*
	12 h	4 (3–5)	3 (2–4)		0.003*
	24 h	4 (4–5)	4 (3–5)		0.395

Data is presented as mean ± SD or median (IQR). *: significant as P value < 0.05. VAS: Visual analog scale

**Fig. 2:**
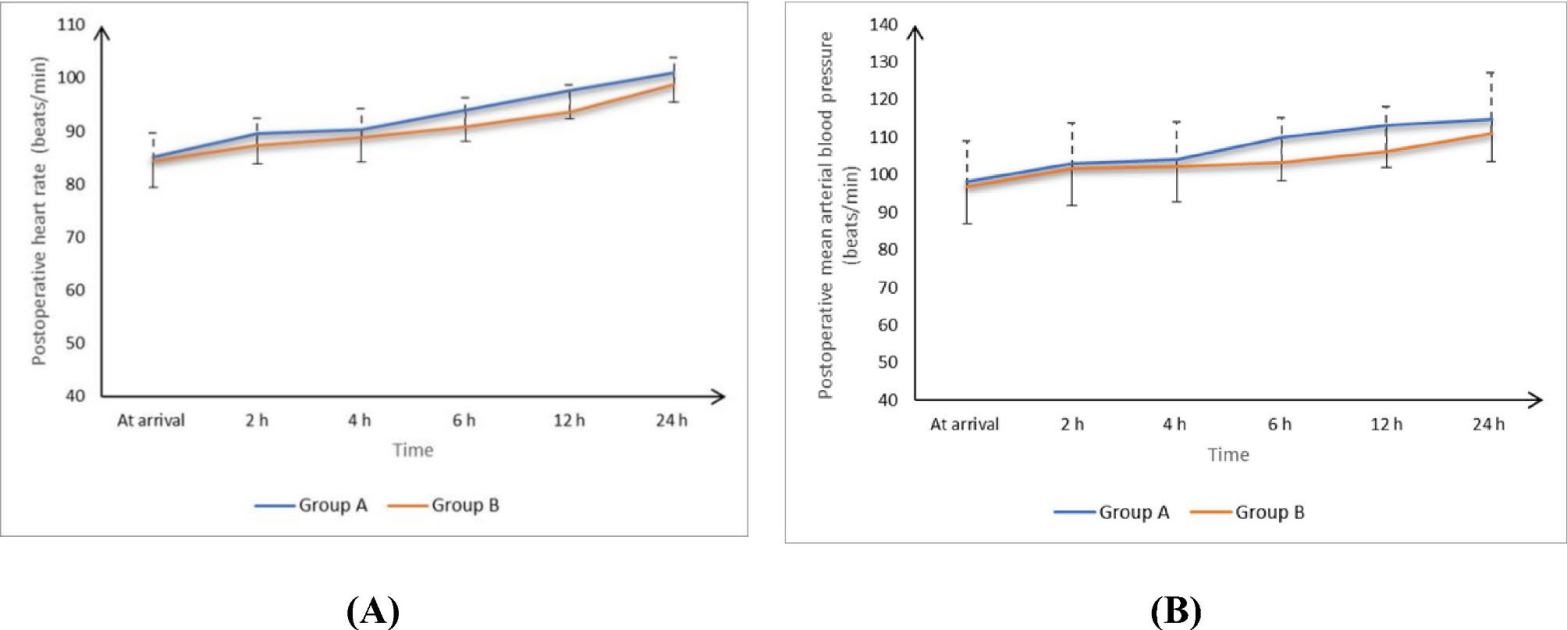
Postoperative (**A**) heart rate and (**B**) mean arterial blood pressure of the two groups

At 6 and 12 h postoperatively, the DB Group had lower HR and MAP than the B Group. On the other hand, neither group differed significantly at arrival, after 2 h, 4 h, or 24 h (Fig. 2). Ramsay sedation score was significantly higher in the DB group at extubation and after 30 min. However, no cases of excessive sedation were observed in the DB group. Neither group demonstrated a statistically significant difference in hypotension (6 patients in the DB Group vs. 3 in the B Group, *p* = 0.278). Neither group experienced local anesthetic toxicity or failed blocks.

## Discussion

This study demonstrated that the addition of dexmedetomidine to bupivacaine during intermediate CPB prolonged the duration of analgesia in patients subjected to thyroidectomy compared to bupivacaine alone. Dexmedetomidine bupivacaine combination significantly reduced postoperative pain, and the total amount of morphine used in the first 24 postoperative hours compared to bupivacaine alone.

In the current study, compared to bupivacaine alone, the latency to the first request for rescue analgesia was significantly longer for the bupivacaine + Dexmedetomidine group. The combination of bupivacaine and dexmedetomidine significantly reduced the overall amount of morphine used in the first twenty-four hours after surgery compared to bupivacaine alone. Similarly to our findings, Elshayeb et al. [10] demonstrated that compared to bupivacaine alone, the combination of 0.25% bupivacaine + 0.5 ml with 50 µg dexmedetomidine significantly delayed the first need for rescue analgesia. The total amount of opioids consumed by the bupivacaine + 0.5 ml plus (50 µg) dexmedetomidine was much lower than bupivacaine only. Supporting our result, El Bendary et al. [11] reported that compared to levobupivacaine alone, the latency to first rescue in levobupivacaine + dexmedetomidine was much longer. Total opioid consumption was significantly lower in levobupivacaine plus dexmedetomidine than in levobupivacaine only.

Comparable to our finding, Hassan et al. [12] revealed that compared to levobupivacine, dexmedetomidine had a much reduced VAS, a much longer duration to first rescue, and much less intake of postoperative analgesics. In line with our result, Elmaddawy and Mazy [13] showed that compared to bupivacaine, dexmedetomidine had a much longer delay before initial rescue. Group D significantly reduced opiate and intraoperative fentanyl intake compared to group B.

In our result, VAS was considerably lower at 6 h and 12 h in bupivacaine + Dexmedetomidine than bupivacaine alone and was insignificantly different at arrival, 2 h, 4 h, and 24 h between the two groups. Elshayeb et al. [10] corroborated our results by revealing that compared to the bupivacaine alone group, the VAS was markedly reduced in the group that received 0.25% bupivacaine in addition to 0.5 ml of dexmedetomidine (50 µg).

El Bendary et al. [11] also revealed comparable outcomes and found that VAS was substantially lower in the levobupivacaine plus dexmedetomidine than the levobupivacaine only. Elmaddawy and Mazy [13] revealed that the VAS was considerably lower in dexmedetomidine than in bupivacaine.

In this study, we observed a decrease in HR and MAP in the dexmedetomidine group at some time points during the postoperative period. This can be attributed to the alpha‑2 adrenoreceptor agonist plus the sedative effects reducing the HR during and after anesthesia. Besides, more patients showed hypotensive episodes in the dexmedetomidine group, however, the difference was not significant (*p* = 0.278). In their investigation, Lin et al. [12] observed increased hypotension, bradycardia, and sedation while administering 1 µg/kg dexmedetomidine. However, stable hemodynamics were attained in most patients with this 1 µg/kg dosage regimen.

Hassan et al. [14], compared dexmedetomidine vs. dexamethasone as an LA adjunct in CPB for patients undergoing thyroid operation. In agreement with the present study, they found significantly lower HR with dexmedetomidine use. However, they did not observe a significant intergroup difference in MAP.

The variation among studies could be attributed to the dose-dependent suppression of MAP and HR by dexmedetomidine, simultaneous with the activation of the vagus nerve. Moreover, the LA diffusion along the carotid sheath may induce vagal blockade, resulting in the reduction of the baroreceptor reflex, subsequent autonomic imbalance, and elevation of blood pressure and heart rate [15].

In addition to its antisympathetic central action, dexmedetomidine activates the vagus nerve, which reduces plasma catecholamine levels and maintains stable hemodynamics by lowering blood pressure and heart rate [12]. The primary mechanism of action of perineural dexmedetomidine is the activation of the sodium-potassium pump, which leads to an increase in membrane hyperpolarization [16].

It is noted that all of these studies adopted the superficial CPB except El Shayeb et al. who used the intermediate technique in some patients. However, the authors did not determine their number or report the results of both techniques separately. In the current study, we preferred the intermediate CPB technique in all patients.

It has been shown that reliance on superficial CPB only for thyroidectomy may be insufficient since the motor and visceral nerves remain unobstructed [17]. On the other hand, deep CPB can lead to significant complications including intravascular injection, epidural or subarachnoid injection, and phrenic nerve palsy [18, 19]. Leblanc et al. [20], described USG intermediate CPB as a straightforward, secure, and dependable procedure.

For implementing the intermediate CPB, the injection should target the posterior cervical space (PCS) lying between the sternomastoid and prevertebral muscles. The superficial branches of the cervical plexus penetrate the prevertebral fascia traversing the PCS and then emerge to the skin and superficial tissues through the posterior border of the sternomastoid muscle [21]. The precise performance of the ultrasound-guided intermediate CPB in the PCS at a particular cervical vertebral level has the potential to simultaneously block all four cutaneous branches of the cervical plexus and sensory/motor branches of the cervical plexus that supply the SCM muscle. This blockade serves to provide sufficient anesthesia and analgesia for different neck surgeries [22]. Many studies reported effective analgesia with intermediate CPB for carotid endarterectomy [23, 24], surgeries involving SCM muscle [22], and cervical esophagus diverticulectomy [25].

Senapathi et al. [26], found that intermediate CPB is superior to the multi-directional subcutaneous injection technique of the superficial CPB in alleviating post-thyroidectomy pain. However, the authors referred to the USG superficial CPB instead of the USG intermediate CPB. This nomenclature discrepancy was also observed in the study of El Bendary et al. [10],. This inconsistency may be attributed to the specific position of the block and the branch of the cervical plexus that is being blocked. A CPB conducted at the cervical paravertebral space (C2–C4) can effectively block both the superficial and deep branches of the cervical plexus in parallel [27]. However, it is referred to as a deep CPB instead of a superficial one, considering the specific location where the block is involved. Hence, it is more suitable to designate the CPB technique used in the PCS, which leads to the intrafacial dissemination of local anesthetic, as an intermediate CPB rather than a superficial CPB [28]. Nevertheless, there remains a possibility of phrenic block, since the LA administered during the intermediate CPB can penetrate profoundly into the phrenic nerve itself [29].

The primary strength and uniqueness of the present study are in the utilization of intermediate, rather than superficial, CPB for patients who are undergoing thyroidectomy specifically for malignant conditions. We examined the additional impact of perineal dexmedetomidine in this particular cohort, providing novel insights into the study of peripheral nerve block in procedures involving the neck area. The limitations of this investigation include the relatively small number of participants and the fact that it was conducted at a single location.

## Conclusions

The addition of dexmedetomidine to bupivacaine in ultrasound-guided intermediate cervical plexus block for thyroidectomy significantly prolonged analgesia and reduced postoperative opioid consumption. The DB group also showed lower heart rate and mean arterial pressure at 6 and 12 h postoperatively. While sedation was higher in the DB group, no excessive sedation or significant hypotension occurred. These findings suggest that dexmedetomidine enhances the effectiveness of the block without major adverse effects. Further research is needed to confirm these results.

## Electronic supplementary material

Below is the link to the electronic supplementary material.

Supplementary Material 1

## Data Availability

Data is available upon reasonable request from the corresponding author.

## Declarations

Informed written consent was obtained from all patients.

## Abbreviations

CPB: Cervical plexus block
α2-AR: Alpha 2 adrenoceptors
ASA: American Society of Anesthesiologists
INR: International normalized ratio
GA: General anesthesia
VAS: Visual Analogue Scale
IV: Intravenous
SCM: Sternocleidomastoid
PONV: Postoperative nausea and vomiting
SD: Standard deviation
IQR: Interquartile range
